# Development of high-performance composite via innovative route using water hyacinth extracted nanocellulose and analysis of its physical properties

**DOI:** 10.1016/j.heliyon.2023.e23095

**Published:** 2023-12-01

**Authors:** Moni Sankar Mondal, Syed Zubair Hussain, Pias Roy, Chanda Halder

**Affiliations:** a Department of Textile Engineering, Khulna University of Engineering & Technology, Khulna, 9203, Bangladesh; b Agronomy and Agricultural Extension, University of Rajshahi, Rajshahi, 6205, Bangladesh; c Department of Physics, University of Rajshahi, Rajshahi, 6205, Bangladesh

**Keywords:** Water hyacinth, Acid hydrolysis & sonication, Nanocellulose, Nanocomposite, Tensile strength, Flexural strength

## Abstract

This study focuses on the development of a high-performance composite using a novel technique incorporating nanocellulose extracted from water hyacinth. The extraction procedure of nanocellulose from water hyacinth stems involves acid hydrolysis and sonication, followed by its incorporation into jute, glass, and cotton fabric through the dip coating method. The crystallinity index of the nanocellulose was determined to be 40.72% using X-ray diffraction (XRD) analysis. Additionally, the functional groups of the extracted nanocellulose were identified through FT-IR analysis, while scanning electron microscopy (SEM) demonstrated morphological changes after nanocellulose coating. Our synthesized water hyacinth nanocellulose exhibited compliance with previously studied results in FT-IR analysis. Both tensile and flexural strength tests revealed that the nanocellulose coating significantly improved the strength of the jute, cotton, and glass fabric-reinforced composites compared to their raw counterparts. Specifically, the jute nanocomposite exhibited a 24.61% increase in strength, the cotton woven nanocomposite showed a 19.39% enhancement, and the glass nanocomposite displayed 8.47% increment in strength. Similarly, the flexural stress of jute and cotton fabric nanocomposites showed a notable 11% and 8.9% increase, surpassing the 3.59% rise observed in glass nanocomposites. Overall, this research successfully completed all tests and achieved superior findings compared to earlier studies.

## 1 Introduction

Nanocellulose has emerged as a promising material in the field of nanotechnology due to its exceptional mechanical properties, renewable nature, biodegradability, and cost-effectiveness [1]. Researchers have successfully extracted nanocellulose from various sources such as kenaf, wheat straw, oil palm empty fruit bunch fiber (OPEFB), pineapple leaf fiber (PLF), Sengkang leaves, leftover sugarcane bagasse, and bacterial cellulose [2]. Additionally, nanocellulose is sourced from both coniferous woods like pine, spruce, and fir, and deciduous woods including oak, maple, and birch [3]. In each case, the removal of lignin and hemicellulose through chemical processes yielded highly refined nanocellulose, typically in the form of a clear colloid solution. Plant-derived nanocellulose can be utilized as a filler in biopolymer composites. With its diameter ranging from 1 to 100 nm, nanocellulose exhibits transparency, low density, and a high contact surface area, along with favorable mechanical properties [4].

Water hyacinth, a commonly found aquatic plant in tropical regions, represents a valuable source of fibers, with cellulose content averaging around 60% [5,6]. However, the cellulose fiber content may vary depending on the plant's growth conditions. Initially, nanocellulose from water hyacinth fibers (WHF) was isolated using cryo-crushing techniques, resulting in diameters of approximately 25 nm and lengths in the micrometer range [7]. Subsequent intense shear homogenization processes led to the formation of aggregate particles with diameters ranging from 25 to 40 nm [8]. Acid hydrolysis, involving the addition of water molecules with the aid of an acid catalyst, is commonly employed to break down chemical compounds into smaller entities [9].

Nanocellulose serves as an excellent reinforcement for polymers, enhancing their properties even at low filler loadings [10]. The incorporation of crystalline nanocellulose with a high aspect ratio and stiffness contributes to the development of materials with improved strength [11]. The high aspect ratio and efficient dispersion of nanocellulose significantly enhance both the mechanical and electrical properties of composite mate [12]. When nanocellulose is combined with acrylic polymers, a novel material with a unique combination of properties derived from its components can be achieved [13]. The resulting material exhibits improved mechanical strength, enhanced stability, reduced weight, and improved thermal stability, making it appealing for various applications such as biomedical, construction, and packaging [14]. This makes the resulting material attractive for a wide range of applications, including biomedical, construction, and packaging [15]. Moreover, the distinctive morphologies imparted by nanocellulose enable tailoring the material's properties to meet specific application requirements [16]. The presence of nanocellulose as a filler leads to the formation of crystalline rod-like particles that hinder the permeation and interpenetration of small molecules and chains at the molecular level [17]. Furthermore, nanocellulose exhibits chemical resistance due to the strong chain-to-chain interactions [18].

The objective of this study is to assess the enhancement of strength in composites reinforced with jute, cotton, and glass fabrics by applying a coating of nanocellulose extracted from water hyacinth. Glass fiber possesses inherent high-strength characteristics [19]. Nevertheless, the incorporation of nanocellulose into glass fiber composites has the potential to reduce processing costs while maintaining a superior level of strength.

The proposed work demonstrate the utilization of water hyacinth which is often considered an environmental nuisance, as a source of nanocellulose for reinforcement in composites. Also, the combination of nanocellulose from water hyacinth with jute, cotton, and glass fabrics has not been previously explored. The incorporation of nanocellulose from water hyacinth into these distinct fabric matrices presents a unique opportunity to investigate synergistic effects and novel material properties that arise from these combinations. The use of water hyacinth for this purpose is innovative and represents a sustainable approach to repurpose a problematic plant into a valuable resource.

If we can develop an industrial demand for water hyacinth, it will become an advantage rather than a burden [20]. To the best of our knowledge, water hyacinth has not been previously utilized as a reinforcing filler in composites. Therefore, our fundamental approach aims to develop natural and synthetic nanocomposites, synthesize nanocellulose from water hyacinth fibers, and demonstrate the sustainability of the resulting composites.

## 2 Materials and methodology

### 2.1 Materials

The WH plants used in this study were collected from a river near Khulna, Bangladesh. Before turning the WH stem into cellulose fiber, the roots and leaves were removed. WHF isolation was carried out using distilled water (H_2_O), sodium chloride (NaCl), sodium hydroxide (NaOH), hydrochloric acid (HCl), and acetic acid (CH_3_COOH). In this study, Bisphenol A based epoxy resin (Araldite LY 556), hardener (Aradur HY 951), and glass fabric (E-glass, 220 GSM) were supplied by Lucky Acrylic and Fiber Ltd., Dhaka, Bangladesh which are presented in Table 1. In this work, Jute fabric (240 GSM) was sourced from Platinum Jute Mills in Khulna, Bangladesh and 100% cotton woven fabric (240 GSM) was received from Apex Holdings Limited (Savar, Bangladesh). All chemical reagents were analytical grade and were collected from the Wet Engineering (WPE) Laboratory at Khulna University of Engineering & Technology in Khulna, Bangladesh.

**Table 1:** Properties of epoxy resin and hardener.

Type	Epoxy resin	Hardener
Mixing proportion	3	1
Specific gravity	1.15 ± 0.1 g/cm^3^	1.02 ± 0.1 g/cm^3^
Color	Colorless	Brown
Viscosity (mPa.s)	10,000–12,000	10–20
Hardening time (h)	24–36 h at 23 °C	

### 2.2 Methodology

#### 2.2.1 Preparation of water hyacinth fiber for nanocellulose extraction

Water hyacinth plants were initially collected from the Rupsha River in Khulna, Bangladesh. Following 72 h of sun-drying, the plants were cut into approximately 1-cm lengths before undergoing chemical treatment. A solution of 15% sodium hydroxide was prepared, and the water hyacinth stems were mixed with it at a ratio of 1:10. Subsequently, the stems were subjected to a high-pressure digester at 5 bar pressure for 6 h at a temperature of approximately 120 °C. The pH of the cooked water hyacinth stems was then adjusted to 7 by immersing them in distilled water, and they were exposed to sunlight for two days until they acquired a paper-like consistency. To create the water hyacinth fiber (WHF) powder, 10 g of dried WHF was mixed with 5% sodium hydroxide (0.5 gm) and heated for 4 h at 60 °C. It is worth noting that this pulping method is significantly less harmful to the environment compared to sulphate pulping techniques.

#### 2.2.2 Bleaching process

The fibers underwent a bleaching process, which involved soaking them in a solution of sodium chlorite and acetic acid at a ratio of 4:1 for 2 h at 60 °C. This treatment effectively transformed the brown color of the water hyacinth fiber (WHF) to white. Subsequently, the bleached fibers were neutralized to a pH of 7 through thorough washing with distilled water. Thorough washing and later FTIR analysis ensured the complete removal of any remaining lignin from the fibers.

#### 2.2.3 Preparation of nanocellulose

**Fig. 1:**
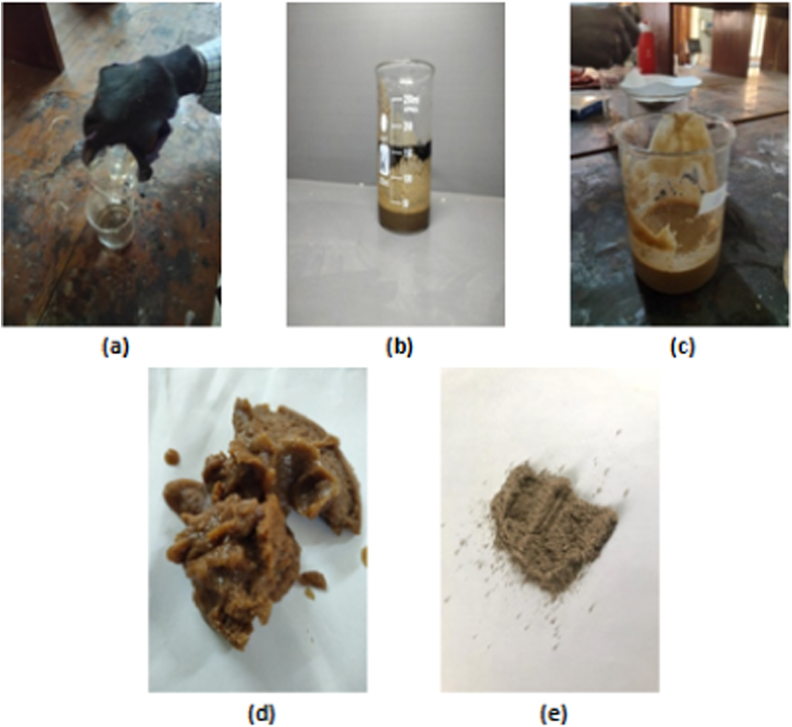
(a) Acid solution, (b) WH cellulose fibers in acid solution, (c) Nanocellulose (liquid), (d) Filtered Nanocellulose, and (e) Nanocellulose powder.

To achieve higher crystallinity, improved uniformity, and smaller water hyacinth (WH) cellulose fibers, acid hydrolysis was conducted in two stages. Initially, the WH cellulose fibers were bleached by treating them with hydrochloric acid (5 M) for 20 h at 60 °C and 500 RPM (Fig. 1(a and b)). Subsequently, a second hydrolysis process was performed using hydrochloric acid (3.5 M) at the same temperature and speed for an additional 20 h. The resulting hydrolyzed fibers were thoroughly washed with distilled water until they reached a neutral pH of 7. To mitigate the harshness associated with the single sulfuric acid hydrolysis method, a double hydrolysis approach was employed to loosen the water hyacinth fiber (WHF) and enhance its responsiveness to sonication. A suspension Fig. 1(c) of WH cellulose (0.5%) in 100 mL of distilled water was subjected to 60 min of sonication below 50 °C using an ultrasonic cell crusher, effectively disintegrating the nanocellulose fibers. The suspension was then dried at 100 °C for 1 h and filtered through a filter paper to remove foreign particles and thus nanocellulose powder was obtained as shown in Fig. 1(d and e).

#### 2.2.4 Preparation of fabric

**Fig. 2:**
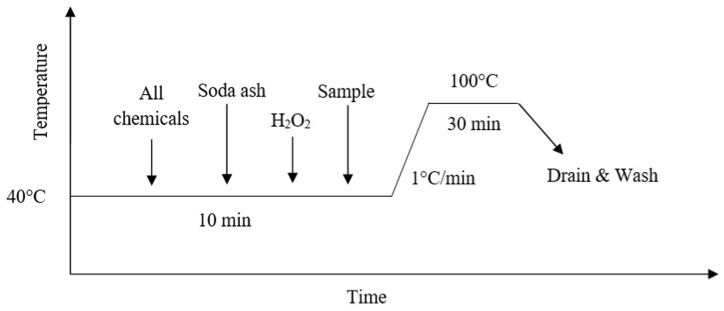
Fabric pretreatment process (Jute and Woven).

Jute fabric was sourced from Platinum Jute Mills in Khulna, Bangladesh, and underwent a pretreatment process to eliminate oil, wax, dirt, dust, and other impurities [13]. For bleaching the sample, of 6 g of soda ash, 2 g/L of detergents, 2 g/L of wetting agent, 2 g/L of stabilizer, and 1 g/L of the sequestering agent along with the sample were taken into a canister and run the sample dyeing machine at 40 °C. After running for 10 min, the temperature was raised to 100 °C maintaining the gradient of 1 °C. After running the machine for 30 min at 100 °C, the samples were taken out from the canister and washed thoroughly using distilled water to eliminate impurities as shown in Fig. 2.

#### 2.2.5 Application of nanocellulose on fabric surface by coating method

At first, 5 gm of the extracted nanocellulose in powder form was introduced into an ethanol solution (50 mL), followed by immersing the fabrics (6 × 6 inch) into the solution. The solution was subsequently heated to 60 °C for 30 min. Following the addition of the binder, the heating process was extended for an additional 30 min. The sample was then taken out of the solution and subjected to padding. Afterward, the samples were dried in a dryer at 100 °C for 1 h, following which they were prepared for further analysis or testing. It was found the GSM of the nanocellulose-induced materials increased. The GSM of Jute, Glass, and Cotton woven fabric was found to be 276, 237, and 270, respectively.

#### 2.2.6 Preparation of composite

**Fig. 3:**
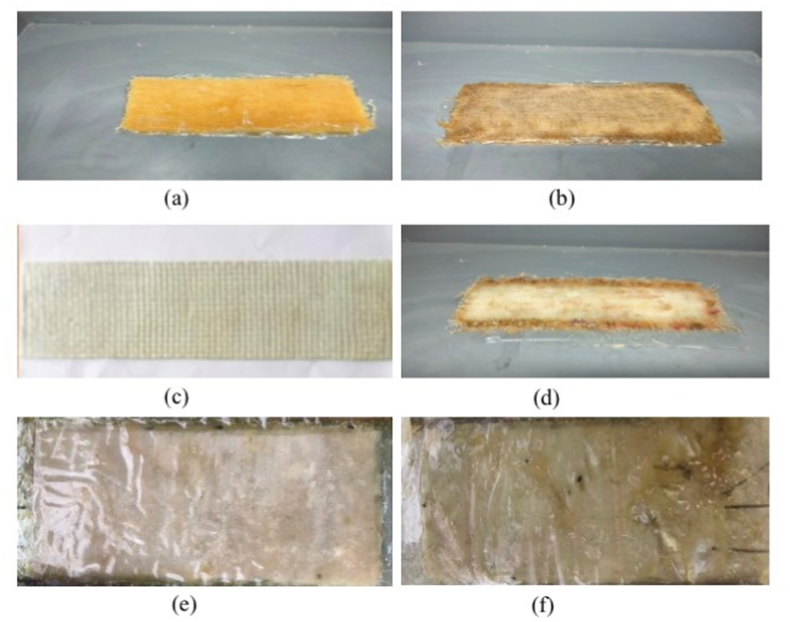
Composite of different composition: (a) Jute, (b) Treated jute, (c) Glass, (d) Treated glass, (e) Cotton woven, and (f) Treated cotton woven fabric composite.

Fig. 3(a–f) shows the developed composite with or without nanocellulose incorporation into Jute, Glass and Woven fabric. As part of the pretreatment process, Jute, Glass, and Woven cloths were cut into rectangular pieces measuring 11 × 4.5 inches. During the composite preparation phase, two different compositions were combined using epoxy resin. A resin-to-hardener ratio of 3:1 was utilized in the hand lay-up method. A ratio of 100 parts by mass of Araldite LY 556, 300 parts by mass of reinforcement fabric (cotton, jute, and glass), and 33 parts by mass of Aradur HY 951 was used and then pressure was applied to obtain the precise rectangular shape of 11 × 4.5 inches composites. The pressure was applied to achieve the desired form, resulting in each sample having an identical form with a thickness of 5 mm.

A total of six composites were created, with three made from raw fabric and the remaining three from fabric coated with water hyacinth nanocellulose. The excess resin was gently squeezed out from the wet composites. Once completely dried, the composites were removed from the mold, and a curing time of 24 h was implemented to achieve optimal hardness and minimize shrinkage. In this study, the weight percentage of the fabric-reinforced composites was adopted as the basis for analysis.

### 2.3 Characterization and measurement

#### 2.3.1 Chemical component analysis

According to a method suggested by the Technical Association of the Pulp and Paper Industry (TAPPI), the amounts of cellulose, hemicellulose, and lignin in the untreated and chemically treated water hyacinth fibers were measured [21].

#### 2.3.2 X-ray diffraction (XRD) analysis of water hyacinth nanocellulose

Before the XRD measurement, the sample was flattened out into the form of a sheet of paper (30 cm × 30 cm × 0.5 cm) and heated at a temperature of 60 °C for 3 h which was then cut into the dimensions of 0.5 cm × 0.5 cm. This ensured that the sample was completely dry and ready for the XRD analysis. X-ray diffraction patterns for Nanocellulose samples were performed on a diffract meter (Shimadzu 600) with Cu Kα radiation (λ = 0.15406 nm) at 40 KV and 30 mA. XRD data were collected from 2θ = 10°–80° at a scan rate of (0.02deg) XRDs of Nanocrystalline cellulose are demonstrated in respective. Similarly, The crystallinity index and average particle size or crystal thickness of nanoparticles was measured using the peak height and Derby Scherer’s equation, respectively, as shown by Equations (1), (2) [13].

(1) $$\text{Crystallinity}\;\text{Index},\text{CrI}=\left[\left(I_{200}-I_{\text{am}}\right)/I_{200}\right]*100\%$$

where I_200_ is the maximum diffraction peak intensity at 2θ = 32.12 (crystalline area). I refer to the diffraction peak at 2θ = 19.04 (amorphous area).

(2) CrystalliteSize(D)=kλ/βcosθ

where k is the shape factor with a value of 0.9, β = 11.85 represents a full-width half maximum of maximum intensity in radians, Bragg angle at peak position, 2θ = 32.12, and λ represents the wavelength of X-ray radiation with the value of 0.15406 nm.

#### 2.3.3 Fourier transform infrared spectroscopy (FTIR)

FTIR analysis (PerkinElmer, USA) was employed to characterize the particles present on the treated fabric. For this analysis, samples were mixed with 2-propanol and compressed into pellets. The transmittance through the functional groups within the size range of 500–4000 cm^−1^ was evaluated as part of the characterization process.

#### 2.3.4 Scanning electron microscopy (SEM)

With a 500–2000× magnification range and a label of 100–400 μm of 15 KV, SEM (FEI INSPECT S50, USA) was employed to examine the surface morphology of the treated samples. This test was conducted at the Bangladesh Atomic Energy Center in Dhaka.

#### 2.3.5 Evaluation of tensile strength of composite

Tensile strength test was done by using a Universal tensile machine (UTM). The purpose of this procedure was to identify the breaking point of the composite samples. According to the ASTM D3039 method, the tensile strength of the composite was tested. At first, composite samples were prepared by cutting them into a rectangular shape of 8" (H) x 2.5" (W), with the environmental conditions maintained at 65% relative humidity (RH) and 25 °C. The sample composites were securely positioned between the upper and lower crossbars of the UTM. Constant rates of load were applied to the testing samples over time to determine their ability to withstand applied tensile forces without breaking or undergoing plastic deformation [22].

#### 2.3.6 Evaluation of flexural strength of composite

The flexural strength test of composite is another important aspect of material characterization, particularly for materials that are subjected to bending or flexing loads [23]. The flexural strength of composite material is characteristic of its ability to endure bending stresses without going through fracture or significant deformation. Using universal testing equipment, the specimen was mounted on two support anvils and subjected to an applied force on one loading anvil in order to determine its properties. This test was conducted to evaluate the materials' durability against bending and to ascertain their flexural strength under different conditions of loading. In this study, ASTM D7264 testing standard was followed [24]. The composite samples were cut into 180 mm × 25 mm along with a thickness of 6 mm and subjected to a load in bending. During testing, a graph demonstrating the values of forces applied to the specimen and the corresponding breaking point of the material was generated on an incline.

## 3 Result and discussion

### 3.1 Chemical components

The primary components of the water hyacinth employed in this study were 61% cellulose, 28% hemicellulose, and 4.4% lignin. Due to changes in the environment and atmosphere, we discovered that the levels of cellulose and hemicellulose were higher than those previously reported, while the low amount of lignin was in good agreement with prior research [[25], [26], [27]].

Surprisingly, after treatment, the cellulose concentration rose to 91.5% while the hemicellulose and lignin percentages dropped dramatically to 7.3 and 0.6%, respectively. Due to the water hyacinth fibers' porosity structure, which made it easier for the alkaline solution to enter, there may have been a significant decrease in the amount of hemicellulose and lignin present [[28], [29], [30]]. The lignin and hemicellulose's -ether linkages and ester bonds were cleaved during the alkaline treatment as a result of the swelling of the fibers, which also caused hemicellulose to become soluble [31].

### 3.2 X-ray diffraction (XRD) analysis of nanocellulose

**Fig. 4:**
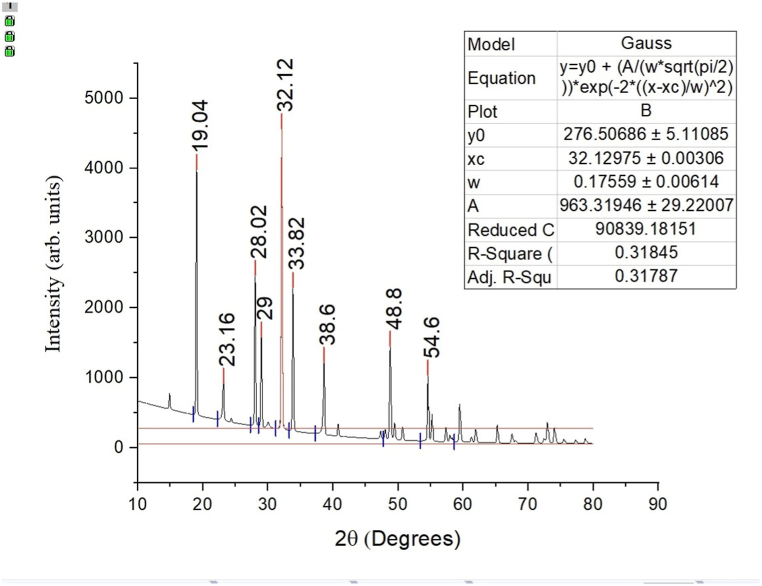
XRD pattern of water hyacinth nanocellulose.

X-ray diffraction (XRD) characterization was conducted to assess the crystallinity index and crystal thickness of the water hyacinth nanocellulose synthesized through acid hydrolysis. Fig. 4 illustrates the XRD features of the nanocellulose, showcasing distinct diffraction peaks representative of a well-defined triclinic crystal system with lattice constants (a = 4.32 Å and c = 4.80 Å). The peaks exhibit increasing sharpness as the scan range (2θ = 5–80°) progresses, with the expansion of the crystalline scale particularly notable at wider angles (2θ). The overall crystallite structure is depicted in Fig. 4.

The water hyacinth nanocellulose exhibits distinct diffraction peaks at specific 2θ angles, namely around 19.04°, 23.5°, 32.12°, and 33.83° corresponding to (102), (200), (040) and (040) crystallographic planes [21,32]. These peaks indicate frequent crystallization and close molecular packing [33].

The acid-treated fiber demonstrates a higher crystallinity index of 40.47% and crystal thickness of 35.95 nm, compared to the untreated water hyacinth fiber with a crystallinity index of 5%. This higher value indicates that the acid treatment penetrated the amorphous portion of the cellulose fiber, leading to its degradation [34].

### 3.3 Fourier transform infrared (FTIR) analysis of nanocellulose

**Fig. 5:**
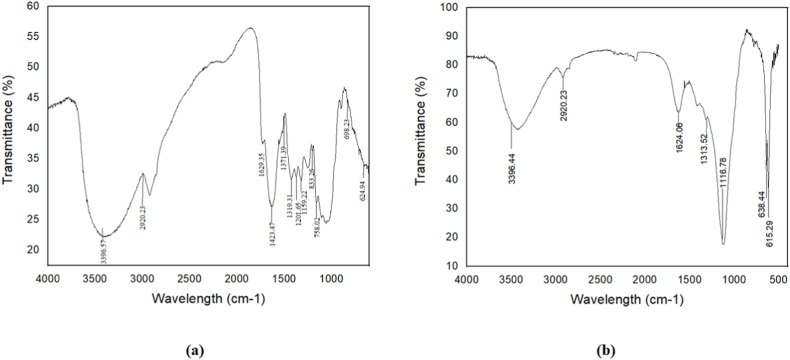
(a) FTIR Spectroscopy of raw cellulose, and (b) FTIR Spectroscopy of WH nano cellulose.

The presence of functional groups in raw cellulose and nanocellulose is analyzed by FTIR and shown in Fig. 5(a) and (b), spanning from a wavelength of 4000 cm^−1^ to 500 cm^−1^. By identifying the functional groups, it is possible to distinguish the amorphous lignin, crystalline cellulose, and hemicellulose. Fig. 5(a) represents the characteristic peak at approximately 3396.44 cm^−1^ corresponds to the stretching of OH bonds resulting from hydrogen interactions with hydroxyl groups. The presence of spectra at 2920.23 cm^−1^ indicates C–H stretching, which can be observed in all samples and suggests the presence of aliphatic saturated components [35].

The absorption peak at 1624.06 cm^−1^ corresponds to the O–H vibration of absorbed water. However, the treated samples showed lower results than expected, potentially due to the breakdown of cellulose chains into smaller, more compact cellulosic molecules with reduced nano-sized porosity [36]. Fig. 5(a) illustrates the aromatic ring vibration of lignin occurring from 1500 to 1400 cm^−1^. The absence of the characteristic peak at 1248 cm^−1^ suggests the successful removal of the lignin component and an increase in the cellulose component due to the chemical treatment, as shown in Fig. 5(b). Additionally, the peaks observed at 1116.78 cm^−1^ in Fig. 5(b) correspond to the stretching vibrations of C–O and C–OH, the out-of-plane bending mode of C–OH, and intermolecular ester bonding.

### 3.4 Scanning electron microscopy

**Fig. 6:**
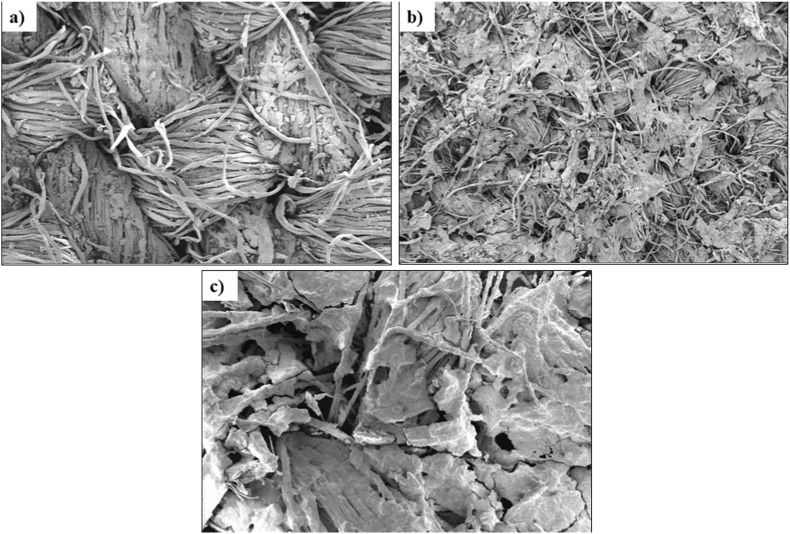
(a) Nanocellulose-coated woven fabric, (b) Nanocellulose-coated jute fabric, and (c) Nanocellulose-coated glass.

The SEM image presented in Fig. 6 provides visual evidence of the presence of nanocellulose on the fabric samples. Specifically, Fig. 6(a), (b), and (c) display the morphological images of the treated glass, woven, and jute fabric, respectively. It is noteworthy that the jute-treated fabric exhibits a significant amount of nanocellulose compared to the other types of treated fabrics. This characterization confirms that the jute fabric possesses a higher surface area, enabling it to display a more pronounced crystalline structure. Furthermore, this finding is consistent with the observed improvement in tensile strength, reinforcing the correlation between the presence of nanocellulose, the enhanced crystalline structure, and the mechanical performance of the jute fabric.

### 3.5 Tensile strength test

**Fig. 7:**
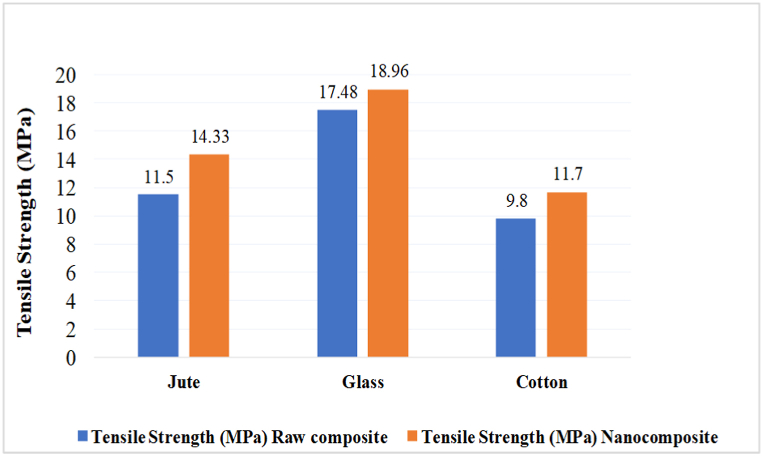
Tensile Strength of individual composite materials.

In Fig. 7, it is evident that the nanocellulose-coated samples exhibited higher tensile strength compared to the uncoated samples. The uncoated glass fabric demonstrated a tensile strength of 17.48 MPa, whereas the nanocellulose-coated glass composite showed an 8.47% increase in strength compared to the raw glass composite. The raw jute fabric composite containing a exhibited a breaking strength of 11.5 MPa, while the nanocellulose-coated jute composite displayed a remarkably 24.61% higher tensile strength than the uncoated counterpart. Similarly, the raw cotton woven fabric sample exhibited a tensile strength of 9.8 MPa, whereas the treated nanocomposite exhibited 11.7 MPa tensile strength, representing a 19.39% increase over the uncoated woven sample.

Precisely, it could be claimed that an increment in tensile strength might be achieved with the coating of nanocellulose compared to their raw counterparts. This improvement is attributed to the reinforcing properties of nanocellulose, which enhance the mechanical characteristics of the composite material due to the high aspect ratio and stiffness of nanocellulose fibers, contributing to increased load transfer and improved stress distribution within the composite structure [37]. Due to its higher surface area, nanocellulose creates a larger contact area and improves the interaction between the nanocellulose coating and the fiber surface [38]. This enhanced interaction leads to the increased interlocking and interlocking forces between the nanocellulose and the jute, woven and glass fiber, contributing to the overall strength of the composite.

### 3.6 Flexural strength test

The flexural strength test helps determine the ability of a material to withstand bending or flexural loads, which are common in structural applications. It provides crucial information about the material's resistance to fracture or deformation under bending stresses, ensuring the structural integrity and reliability of the material.

**Fig. 8:**
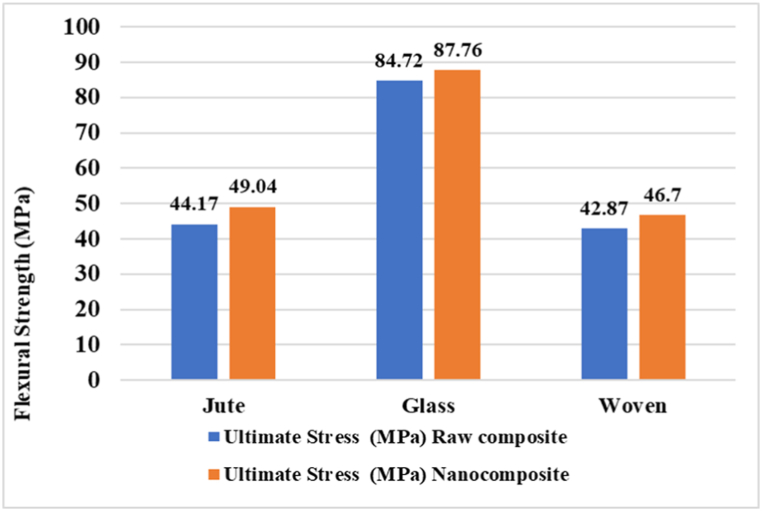
Flexural strength of individual composite materials (raw and nanocelluolose coated composite).

Based on Fig. 8, it is evident that both the nanocellulose-coated Jute and Woven fabric samples display comparable flexural strength values. Jute nanocomposite has a flexural strength of 49.04 MPa, while cotton nanocomposite has a flexural strength of 46.7 MPa, both of which are higher than their raw counterparts. On the other hand, both the raw and nanocomposite forms of Glass demonstrate higher flexural strength compared to the Jute and Woven fabric composites. Notably, the glass nanocomposite exhibits a 3.59% increase in flexural strength compared to the raw glass composite, whereas the jute and cotton fabric nanocomposite show an 11% and 8.9% increment in flexural strength, respectively. This is due to the less amount of nanocellulose present on the surface of glass fiber does not enhance the interfacial bonding between the fiber and the matrix material as jute and cotton fabric. This improved interfacial adhesion of jute and cotton results in efficient load transfer between the fiber and the matrix, reducing the likelihood of fiber-matrix debonding or failure than nanocellulose-coated glass fabric composite [39].

This result confirms that the highly coated nanomaterials on the fabric would be able to absorb more loads without failure due to more interfacial bonding strength and would comply with the findings of morphological analysis by SEM of the treated fabrics [40].

## 4 Conclusion

Based on the research findings, the nanocellulose-based jute, cotton woven, and glass fabric epoxy composites demonstrate superior mechanical properties in comparison to the raw jute and glass fabric composites. This could be particularly valuable in creating stronger and more durable textiles for clothing, upholstery, and technical textiles used in industries like automotive and aerospace. Also the improved strength and potentially unique properties of the nanocellulose-coated textiles can be used in biodegradable packaging materials, especially for products that require robust packaging solutions. Consequently, these composites are highly suitable for industrial applications in workwear, military uniforms, and other clothing where durability is important.

The key outcomes of the study can be summarized as follows:

1. Successful extraction of high-purity nanocellulose in a nanometer-sized form from Water Hyacinth Fiber (WHF) using acid hydrolysis. This achievement is supported by observations from XRD and FTIR analyses. The hydrolysis process resulted in a remarkable crystal thickness (35.95 nm) and crystallinity index (40.72%).
2. The composites with nanocellulose exhibited enhanced tensile strength. Specifically, the tensile strengths of the jute, cotton, and glass fabric nano-composite were reported as 14.33 MPa, 11.7 MPa, and 18.96 MPa, respectively. The nanocomposite of jute exhibited a 24.6% increase in tensile strength compared to the raw composite, while the glass nanocomposite displayed an 8.47% increase in tensile strength. Additionally, the nanocomposite of cotton woven fabric demonstrated a 19.38% enhancement in tensile strength compared to the raw composite.
3. The nanocomposites exhibited satisfactory flexural stress values. Specifically, the flexural stress was found to be 49.04 MPa, 47.76 MPa, and 46.7 MPa, for jute, cotton, and glass fabric nanocomposite, respectively. The inclusion of nanocellulose enhances the flexural stress of the composites by improving the interfacial bonding between the fiber and the matrix material. Notably, the nanocellulose-coated jute and cotton fabric-epoxy composites demonstrated an increase in flexural stress of 11% and 8.93%, respectively, compared to the raw counterparts. Furthermore, the nanocellulose-coated glass fabric-epoxy composite exhibited a 3.59% higher flexural stress compared to the raw composite.

In summary, the utilization of nanocellulose in the fabrication of composite materials led to improved mechanical properties, making these composites highly desirable for various industrial applications. Nevertheless, this research was conducted using a standard sample size in a laboratory setting. As a result, potential challenges could arise when attempting to transition to large-scale manufacturing due to concerns regarding maintaining consistent quality, ensuring cost-effectiveness, and establishing feasibility at an industrial scale.

## Funding statement

This research did not receive any specific grant from funding agencies in the public, commercial, or not-for-profit sectors.

## Data availability statement

All data generated or analyzed during this study are included in this research article.

## CRediT authorship contribution statement

**Moni Sankar Mondal:** Writing – review & editing, Writing – original draft, Visualization, Validation, Supervision, Resources, Project administration, Methodology, Investigation, Formal analysis, Data curation, Conceptualization. **Syed Zubair Hussain:** Writing – original draft, Resources, Methodology, Data curation. **Pias Roy:** Resources, Methodology, Investigation, Formal analysis. **Chanda Halder:** Resources, Project administration, Methodology, Investigation.

## Declaration of competing interest

The authors declare that they have no known competing financial interests or personal relationships that could have appeared to influence the work reported in this paper.
